# Dipeptidyl peptidase-4 cell surface expression marks an abundant adipose stem/progenitor cell population with high stemness in human white adipose tissue

**DOI:** 10.1080/21623945.2022.2129060

**Published:** 2022-10-03

**Authors:** Florian M Hatzmann, Sonja Großmann, Petra Waldegger, G Jan Wiegers, Markus Mandl, Tina Rauchenwald, Gerhard Pierer, Werner Zwerschke

**Affiliations:** a Division of Cell Metabolism and Differentiation Research, Research Institute for Biomedical Aging Research, University of Innsbruck, Innsbruck, Austria; b Center for Molecular Biosciences Innsbruck, University of Innsbruck, Innsbruck, Austria; c Division of Developmental Immunology, Biocenter, Medical University Innsbruck, Innsbruck, Austria; d Department of Plastic and Reconstructive Surgery, Innsbruck Medical University, Innsbruck, Austria

**Keywords:** Adipogenesis, CD26, DPP4, *ex vivo*, human adipose stem cells, progenitors, proliferation, quiescence, stemness, self-renewal

## Abstract

The capacity of adipose stem/progenitor cells (ASCs) to undergo self-renewal and differentiation is crucial for adipose tissue homoeostasis, regeneration and expansion. However, the heterogeneous ASC populations of the adipose lineage constituting adipose tissue are not precisely known. In the present study, we demonstrate that cell surface expression of dipeptidyl peptidase-4 (DPP4)/cluster of differentiation 26 (CD26) subdivides the DLK1^−^/CD34^+^/CD45^−^/CD31^−^ ASC pool of human white adipose tissues (WATs) into two large populations. *Ex vivo*, DPP4^+^ ASCs possess higher self-renewal and proliferation capacity and lesser adipocyte differentiation potential than DDP4^−^ ASCs. The knock-down of DPP4 in ASC leads to significantly reduced proliferation and self-renewal capacity, while adipogenic differentiation is increased. Ectopic overexpression of DPP4 strongly inhibits adipogenesis. Moreover, in whole mount stainings of human subcutaneous (s)WAT, we detect DPP4 in CD34^+^ ASC located in the vascular stroma surrounding small blood vessels and in mature adipocytes. We conclude that DPP4 is a functional marker for an abundant ASC population in human WAT with high proliferation and self-renewal potential and low adipogenic differentiation capacity.

## Introduction

White adipose tissues (WATs) are major triglyceride storage depots possessing important metabolic and endocrine functions [1]. These physiological functions are mainly fulfilled by adipocytes, the specialized cell type in WAT, which arise out of adipose stem/progenitor cells (ASCs) within the adipogenic lineage [2]. ASCs are essential for adipose tissue homoeostasis, regeneration [3–6] and expansion [7], and, hence, contribute to the maintenance of metabolic health. Their functional decline in obesity and ageing plays most likely an important part in the development of age-associated diseases [8–12].

The majority of ASCs in human WAT reside at the vascular interface surrounding small blood vessels [13–16] and exist in a quiescent state, expressing high levels of the somatic stemness factors cellular-myelocytomatosis oncogene (c-MYC) and Kruppel-like factor 4 (KLF4) and the early adipogenic transcription factor CCAAT/enhancer-binding protein β (C/EBPβ) [17]. According to the current model, c-MYC and KLF4 contribute to the balancing of quiescence, proliferation and differentiation at a hub at which growth-arrested stem/progenitor cells are either directed towards cell cycle entry or committed to terminal differentiation [18–20]. c-MYC facilitates proliferation and inhibits differentiation, while KLF4 acts as a negative regulator of proliferation and an activator of C/EBPβ. After appropriate stimulation, adipogenesis modulating signal transduction pathways eventually activate the expression of the adipogenic key factor peroxisome proliferator-activated receptor-γ 2 (PPARγ2) and additional transcription factors, including members of the C/EBP family, and the terminal adipocyte differentiation programme becomes activated [21,22].

ASCs are routinely isolated from the stromal vascular fraction (SVF) of WAT [23–26]. Sorting of SVF cells for surface staining of the stem cell markers Delta-like protein 1 (DLK1)/Preadipocyte factor-1 (Pref-1) [27,28] and Cluster of differentiation 34 (CD34) [29,30] identifies three main subpopulations: DLK1^+^/CD34^−^, DLK1^+^/CD34^dim^ and DLK1^−^/CD34^+^ cells [14]. Only the latter has a DLK1^−^/CD34^+^/CD90^+^/CD45^−^/CD31^−^ immune phenotype and exhibits proliferative and adipogenic differentiation capacity [14,17]. This ASC population accounts for approximately 50% of all SVF cells and contains the major ASC fraction; however, it still consists of heterogeneous cell types, most likely including specific ASC types, progenitor cells and regulatory stromal cells [12,31]. Further progress in the characterization of the ASC pool should lead to the identification of specific stages in the hierarchy of the adipose lineage. In fact, it was shown that CD24, a glycosylphosphatidylinositol-linked cell surface receptor [32] that plays a role in the regulation of proliferation and differentiation in several stem cell types [33,34] and tracks pluripotent states in mouse and human cells [35], further subdivides the human DLK1^−^/CD34^+^/CD90^+^/CD45^−^/CD31^−^ ASC population in cell surface CD24^+^ and CD24^−^ cells [17]. CD24^+^ ASCs in both mouse [4,36] and human WAT [17] possess a high grade of stemness and give rise to CD24^−^ cells that are closer to a preadipocyte stage. Another interesting candidate for a stemness marker is Dipeptidyl peptidase-4 (DPP4)/CD26, a cell surface protein that has been shown to regulate the fate of haematopoietic stem/progenitor cells as well as more mature blood cells [37–40]. DPP4 is upregulated in the course of adipogenesis and has adipokine-like functions in mature adipocytes [41–44]. More recently, Merrick et al. 2021 [45] reported DPP4 as a surface marker for a rare proliferation competent and multipotent ASC population with relatively low adipogenic differentiation capacity in WAT of mice. However, the importance of DPP4 in human ASC is little studied so far. The aim of the present study is to better understand the role of DPP4 in human ASC.

## Results

### DPP4^+^ cells are a highly abundant fraction of the DLK1^−^/CD34^+^ ASC population in human sWAT

**Figure 1.**
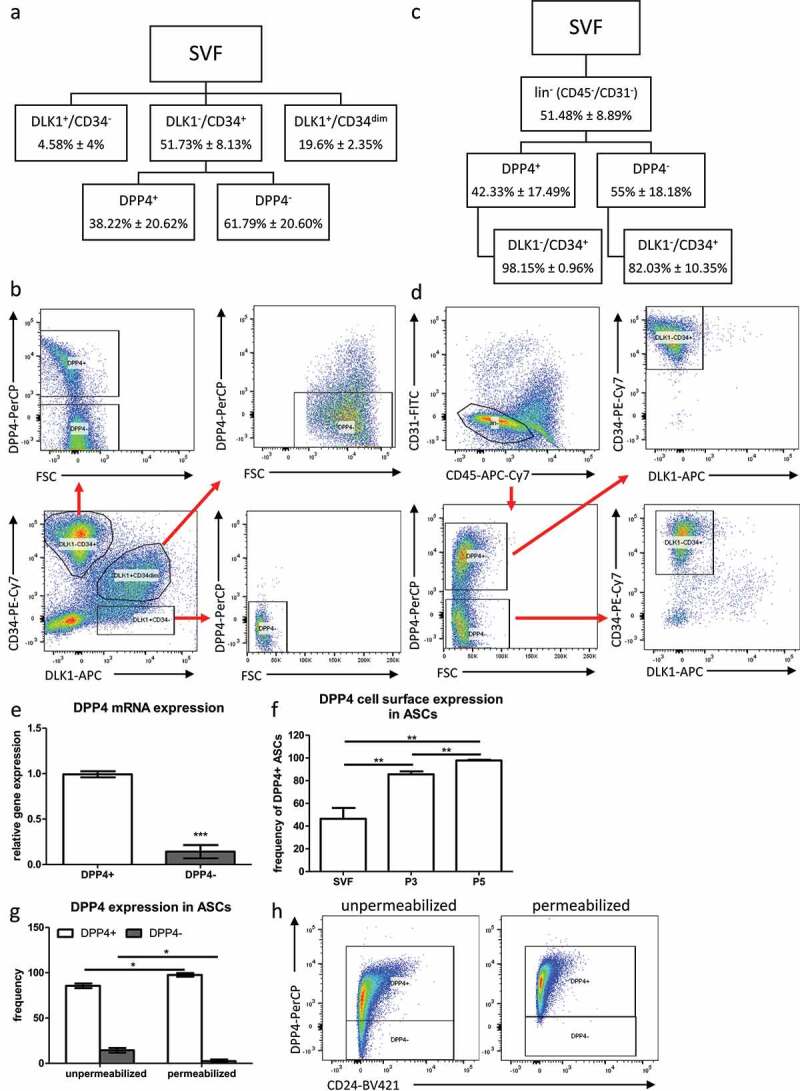
**DPP4 expression in DLK1^−^/CD34^+^, DLK1^−^/CD34^dim^ and DLK1^+^/CD34^−^ populations**. (a) Sorting scheme and (b) representative dot blots showing gating for DPP4^+^ and DPP4^−^ cells in DLK1^−^/CD34^+^, DLK1^−^/CD34^dim^ and DLK1^+^/CD34^−^ populations of the SVF. (c) Sorting scheme and (d) representative dot blots showing gating for DPP4^+^ and DPP4^−^ cells in CD45^−^/CD31^−^ (lin^−^) SVF. *n* = 6 donors, mean ± SD is shown. (e) Relative DPP4 gene expression assessed by RT-qPCR in DLK1^−^/CD34^+^/DPP4^+^ and DLK1^−^/CD34^+^/DPP4^−^ sorted ASC. (f) Frequency of DPP4^+^ cells upon *in vitro* cultivation in the SVF (passage −1), and passage 3 and passage 5 ASC after standard ASC isolation by plastic-adherence. (g) Frequency of DPP4^+^ and DPP4^−^ cells in unpermeabilized and permeabilized passage 3 ASC and (h) representative dot blots. *n* = 3 donors, gene expression was normalized to β-actin, mean ± SEM is shown.

To characterize the DPP4 status of ASC in human sWAT, we analysed cell populations defined by the cell surface proteins DLK1, CD34 and DPP4 *ex vivo*. To do this, freshly isolated SVFs from sWAT were immediately subjected to fluorescence-activated cell sorting (FACS) analysis of DLK1^+^/CD34^−^, DLK1^+^/CD34^dim^ and DLK1^−^/CD34^+^ populations (Figure 1a, 1b). DLK1^−^/CD34^+^ cells are also positive for the stromal cell marker CD90 and negative for the haematopoietic marker CD45 and the endothelial marker CD31 (DLK1^−^/CD34^+^/CD90^+^/CD45^−^/CD31^−^) (Figure 1c, 1d), as shown previously [14,17]. Only the DLK1^−^/CD34^+^ cell population displays high self-renewal, proliferative and adipogenic differentiation capacity [14,17]. Further analysis of the three DLK1/CD34-defined populations reveals that the DLK1^−^/CD34^+^ population contains the vast majority of DPP4^+^ cells, while the DLK1^+^/CD34^−^ population contains only few DPP4^+^ cells and the DLK1^+^/CD34^dim^ population consists exclusively of DPP4^−^ cells. With approximately 46.43% ± 19.15% of the DLK1^−^/CD34^+^ cells being DPP4^+^ and 51.25% ± 19.73% being DPP4^−^, both populations are approximately equally distributed (Figure 1a, 1b) albeit relatively high donor dependent variations were detected (Table 1). Additionally, we analysed cell surface expression of DPP4 in the context of the haematopoietic marker CD45 and the endothelial marker CD31. When we subdivide the CD45^−^/CD31^−^ (lin^−^) population of the SVF into DPP4^+^ and DPP4^−^ cells and subsequently analyse the expression of DLK1 and CD34, we find that the DPP4^+^ population consists almost exclusively (98.15% ± 0.96%) of DLK1^−^/CD34^+^ cells, and the majority (82.03% ± 10.35%) of cells in the DPP4^−^ population are DLK1^−^/CD34^+^ (Figure 1c, 1d, Table 1). Together, these data suggest that CD31^−^/CD45^−^/DPP4^+^ cells in the SVF are a major subpopulation of DLK1^−^/CD34^+^ ASC and are not detectable and little abundant in DLK1^+^/CD34^−^ and DLK1^+^/CD34^dim^ populations, respectively.

**Table 1.** Frequency of DPP4^+^ and DPP4^−^ ASCs in single donors.

	% DPP4^+^	% DPP4^−^
Donor 7	38.60	61.36
Donor 8	23.24	76.81
Donor 9	54.75	45.32
Donor 10	65.20	34.83
Donor 17	8.25	91.74
Donor 18	39.28	60.66
Mean	38.22	61.79
SD	20.62	20.60

Further analyses showed that the small DLK1^−^/CD34^+^/CD24^+^ ASC population with high stemness and low adipogenic capacity [17] is almost exclusively found in the DPP4^+^ ASC population (Supplementary Fig. S1). Gene expression analysis in DLK1^−^/CD34^+^/DPP4^+^ and DLK1^−^/CD34^+^/DPP4^−^ ASC directly after sorting shows that DPP4 mRNA levels are significantly lower in DPP4^−^ ASC (Figure 1e) albeit still detectable, suggesting that these cells have a basal expression of DPP4 and contain most likely intracellular DPP4 protein. Routinely, by plastic adherence, isolated ASCs show a progressive increase in the cell surface DPP4^+^ subpopulation upon *in vitro* cultivation (Figure 1f). The proportion of DPP4^+^ ASCs increases to about 85% in passage 3 and almost 100% in passage 5 (Figure 1f), although the analysis of permeabilized ASCs shows that all ASCs contain DPP4 protein intracellularly as shown for passage 3 ASC (Figure 1g, 1h).

In summary, our data suggest that *ex vivo* sorted cell surface DPP4^+^ cells constitute a major ASC population.

### DPP4 protein can be detected in ASC and mature adipocytes of human WAT

**Figure 2.**
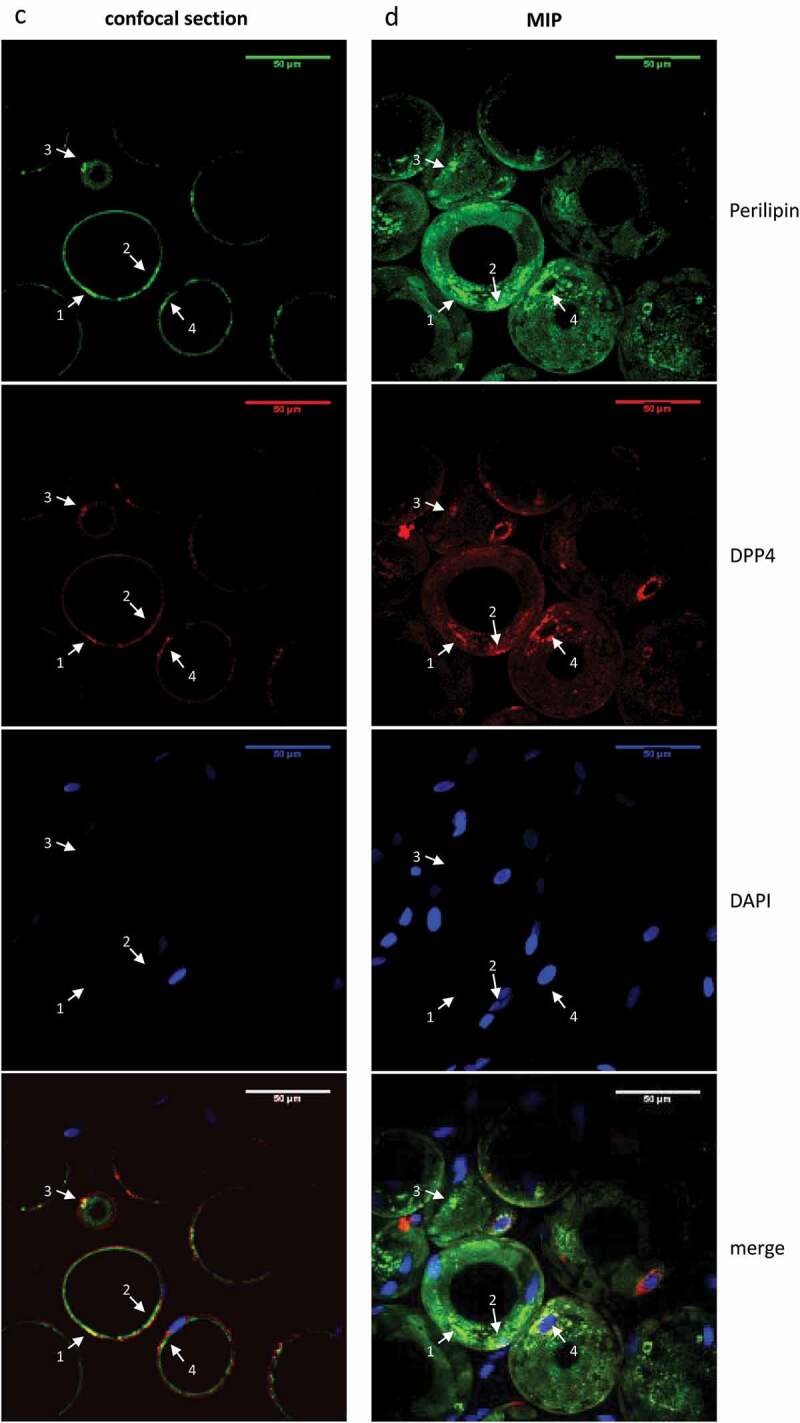
(Continued).

We employed whole mount staining to analyse the localization of DPP4^+^ cells in human sWAT (Figure 2). This analysis shows that DPP4 protein is localized in CD34^+^ cells residing in the vascular interface around small blood vessels (Figure 2a, 2b, arrowheads 1 and 2), the typical niche of ASC in sWAT [13,14,16]. CD34^+^ cells lacking DPP4 protein were also detected in this niche (Figure 2a, 2b, arrowhead 3). DPP4 is also detectable in Perilipin^+^ mature adipocytes, where it is tethered to the adipocyte cell surface (Figure 2c, 2d, arrowheads 1–3) and localized intracellularly in structures surrounding the nuclei, most likely the ER (Figure 2c, 2d, arrowhead 4).
**Figure 2.**
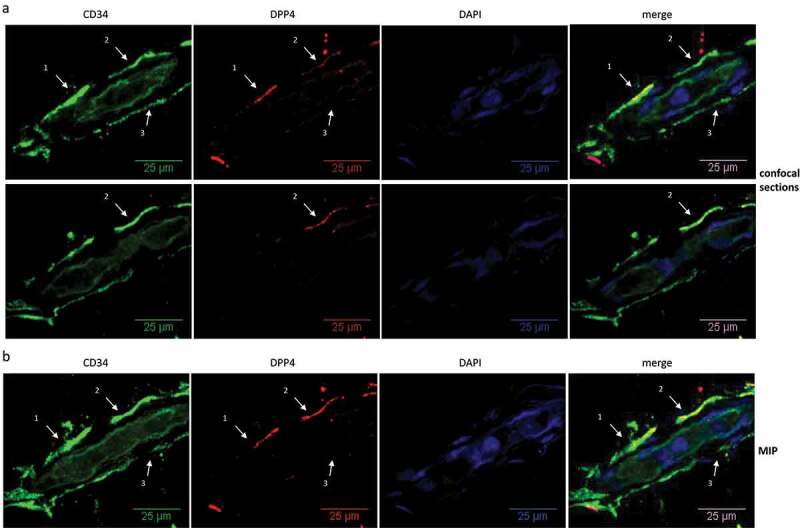
**Localization of DPP4^+^ cells in human sWAT**. Whole mount IF staining of human sWAT analysed by confocal microscopy. (a, b) CD34 (green), DPP4 (red) and nuclei (blue) staining of a small blood vessel. (a) Two diagonal section planes with a distance of 6 µm between each. (b) Maximum intensity projection (MIP) of all sections (3D structure). Wavy CD34^+^ (arrow heads 1–3) and CD34^+^/DPP4^+^ cells (arrow heads 1 and 2) in the perivascular layer around the blood vessel are shown. (c, d) Perilipin (green), DPP4 (red) and nuclei (blue) staining of adipocytes. (c) One diagonal section plane and (d) MIP of all sections (3D-structure), showing DPP4 staining on the cell surface (arrow heads 1–3) and intracellularly in structures surrounding the nuclei (arrow head 4) of unilocular adipocytes.

### Cell surface DPP4^+^ ASCs possess higher proliferative and self-renewal capacity and lower adipogenic differentiation capacity than DPP4^−^ ASCs ex vivo

**Figure 3.**
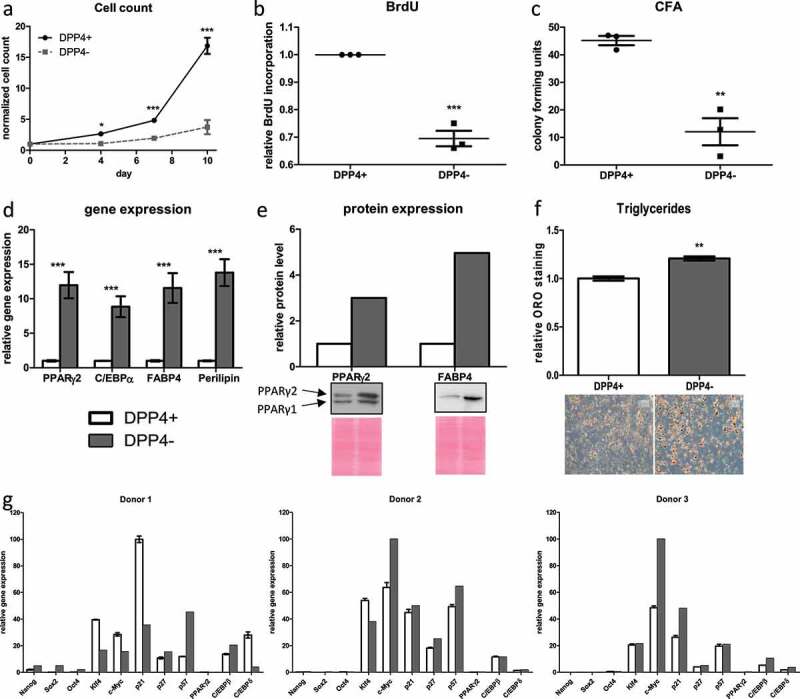
**Cell surface DPP4^+^ ASCs possess higher proliferative and self-renewal capacity but lower adipogenic capacity than DPP4^−^ ASCs *ex vivo.*** Directly after isolation, SVFs were subjected to flow cytometric sorting to compare DPP4^+^ and DPP4^−^ DLK1^−^/CD34^+^ ASC. (a) Growth curve assessed as a number of events recorded in 90 s with FACS, (b) BrdU incorporation assay and (c) colony formation assay were performed as a measure of proliferative and self-renewal capacity; *n* = 3 donors, mean ± SEM is shown. Adipogenic differentiation was assessed by the analysis of (d) gene expression of PPARγ2, C/EBPα, FABP4 and Perilipin and (e) protein levels of PPARγ2 and FABP4 on day 9, and (f) triglyceride formation on day 14 post-induction of adipogenesis, representative results from three donors are shown, mean ± SEM is shown. (g) Gene expression analysis of NANOG, SOX2, OCT4, KLF4, c-MYC, p21^Cip1^, p27^Kip1^, p57^Kip2^, PPARγ2, C/EBPβ and C/EBPδ of three donors immediately after sorting; for analysis, gene expression was normalized to the highest expressed gene which was set to 100. Gene expression was normalized to β-actin, and protein expression was normalized to total protein via Ponceau S staining.

Directly after isolation, *ex vivo*, SVFs from different donors were sorted into cell surface DPP4^+^ and DPP4^−^ ASC subpopulations and subjected to functional analyses. In these experiments, DPP4^+^ cells show considerably higher proliferative capacity than DPP4^−^ cells as analysed by growth curves (Figure 3a) and BrdU incorporation (Figure 3b). Self-renewal capacity, assessed by colony formation assay, is significantly higher in DPP4^+^ relative to DPP4^−^ ASC (Figure 3c). Given the relatively low proliferative capacity of DPP4^−^ ASC, we suppose that in the bulk of ASCs routinely isolated by plastic-adherence proliferation competent, DPP4^+^ ASCs most likely overgrow DPP4^−^ ASCs upon in vitro passaging leading to increasing prevalence of DPP4^+^ ASCs in higher passages (Figure 1f). DPP4^−^ ASCs undergo adipogenic differentiation more effective than DPP4^+^ ASCs as shown by increased expression of the adipogenic marker genes PPARγ2, C/EBPα, FABP4 and Perilipin (Figure 3d), as well as higher protein levels of PPARγ2 and FABP4 upon induction of adipogenesis (Figure 3e). Moreover, premature adipocytes developed from DPP4^−^ ASC contain higher amounts of triglycerides (Figure 3f).

Gene expression analysis directly after sorting reveals that none of the pluripotency markers NANOG, SOX2 or OCT4 is considerably expressed neither in DPP4^+^ nor DPP4^−^ ASC (Figure 3g). However, the somatic stemness markers KLF4 and c-MYC as well as the quiescence markers p21^Cip1^, p27^Kip1^ and p57^Kip2^ are highly expressed in both populations only with donor-dependent variations between DPP4^+^ and DPP4^−^ ASC. While we can hardly detect the adipogenic key regulator PPARγ2, the expression of C/EBPβ and C/EBPδ is detectable in both cell populations (Figure 3g).

Taken together, our data suggest that DPP4^−^ ASCs comprise an ASC population in human sWAT (Figure 1a, 1b) with significantly lower proliferative and self-renewal capacity but a higher adipogenic differentiation capacity than DPP4^+^ ASCs (Figure 3a–3f). Both DPP4^+^ and DPP4^−^ ASCs show the typical gene expression pattern of stemness and quiescence marker of ASC in human sWAT [17].

### DPP4 is required for proliferation and self-renewal of human ASC

**Figure 4.**
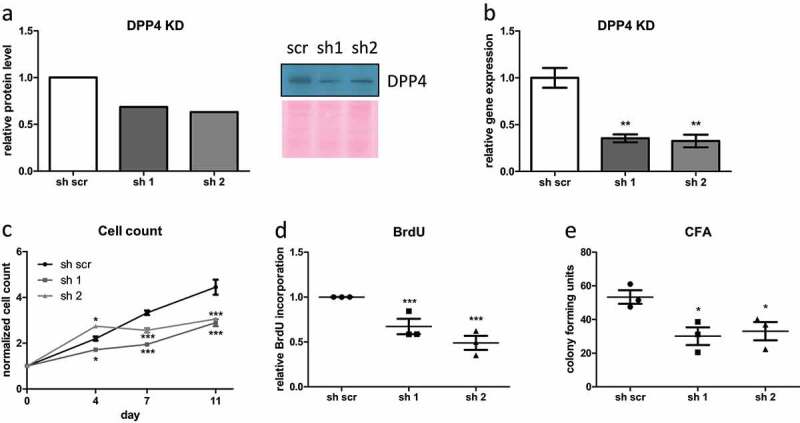
**DPP4 is required for proliferation and self-renewal of human ASC**. shRNA-mediated DPP4 KD in human ASC was generated and confirmed on (a) protein level and (b) gene expression level (sh1 = anti-DPP4 shRNA1, sh2 = anti-DPP4 shRNA2, sh scr = scrambled control shRNA). (c–e) In DPP4 KD ASC, (c) growth curve was assessed as a number of events recorded in 90 s with FACS, (d) BrdU incorporation assay and (e) colony formation assay was performed as a measure of proliferative and self-renewal capacity. (a, b) representative results from three donors are shown. (c–e) *n* = 3 donors, mean ± SEM is shown. Gene expression was normalized to β-actin, and protein expression was normalized to total protein via Ponceau S staining.

To evaluate whether loss-of-function of DPP4 affects ASC proliferation, DPP4 was knocked down (KD) in routinely isolated ASC by RNA interference using gene-specific shRNA (Figure 4a, 4b). The DPP4 KD significantly reduced proliferation rate as analysed by cell count (Figure 4c) and BrdU incorporation (Figure 4d). Moreover, self-renewal capacity was significantly reduced in DPP4 KD ASC, as analysed by colony formation assay (Figure 4e).

### DPP4 negatively regulates adipogenic differentiation of human ASC

**Figure 5.**
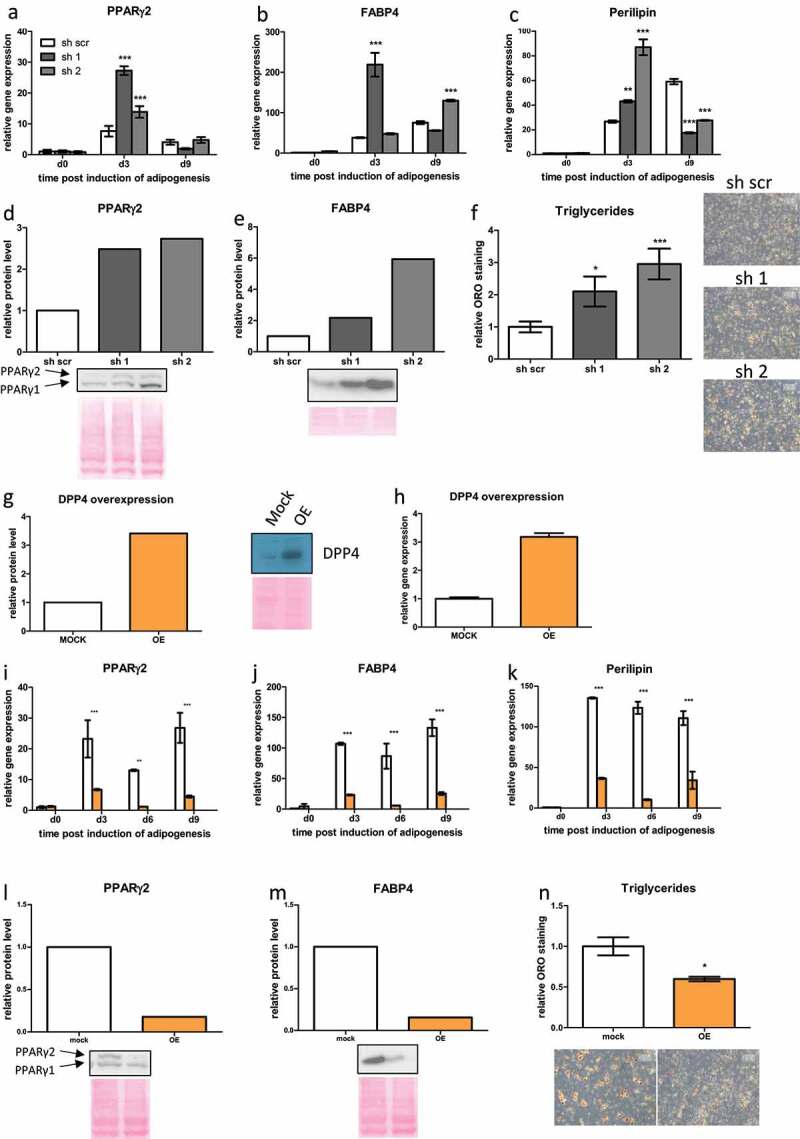
**DPP4 negatively regulates adipogenic differentiation of human ASC**. (a–f) In DPP4 KD ASC, adipogenic differentiation was assessed by the analysis of gene expression of (a) PPARγ2, (b) FABP4 and (c) Perilipin as indicated. Moreover, protein levels of (d) PPARγ2 and (e) FABP4 were measured at day 9, and (f) triglyceride accumulation on day 14 post-induction of adipogenesis. (g–n) Analysis of protein level of adipogenic marker proteins in the course of adipogenesis in DPP4 overexpressing (OE) ASC. (g, h) DPP4 overexpression (OE) in ASC confirmed on (g) protein level and (h) gene expression level. (i–n) In DPP4 OE ASC, adipogenic differentiation was assessed by the analysis of gene expression of (i) PPARγ2, (j) FABP4 and (k) Perilipin as indicated. Moreover, protein levels of (l) PPARγ2 and (m) FABP4 were measured at day 9 and (n) triglyceride accumulation on day 14 post-induction of adipogenesis. Representative results from three donors are shown, mean ± SEM is shown, gene expression was normalized to β-actin and protein expression was normalized to total protein via Ponceau S staining.

Finally, we analyse adipogenic differentiation in DPP4 KD ASC. We find that adipogenic differentiation is significantly increased in ASC upon DPP4 KD, as shown by elevated expression of the adipogenic marker genes PPARγ2, FABP4 and Perilipin, especially on day 3 post-induction of adipogenesis (Figure 5a-5c), upregulated protein levels of PPARγ2 and FABP4 on day 9 (Figure 5d, 5e) and increased triglyceride levels (Figure 5f) on day 14 post-induction of adipogenesis. Together these data suggest that DPP4 KD activates terminal adipogenic differentiation in human ASC. In contrast, ectopic overexpression (OE) of DPP4 in ASC (Figure 5g, 5h) results in significantly reduced adipogenic differentiation capacity (Figure 5i–5n), as shown by dramatically reduced gene expression of PPARγ2, FABP4 and Perilipin (Figure 5i–5k), reduced PPARγ2 and FABP4 protein levels on day 9 (Figure 5l, 5m) and lower triglyceride levels on day 14 post-induction of adipogenesis (Figure 5n).

To summarize this part, experiments inducing loss-of-function and gain-of-function of DPP4 in ASC suggest that DPP4 is required for the maintenance of proliferation and self-renewal capacity and attenuation of adipogenic differentiation.

## Discussion

Adipose tissue contains populations of adult stem and progenitor cells in its SVF that possess the capacity to self-renewal and develop into adipocytes [3–5,23]. These cells are referred to as ASC or mesenchymal stromal cells and belong to the adipose lineage, which is necessary for adipose tissue homoeostasis, regeneration and expansion [46]. ASCs are comprised of distinct subpopulations with different characteristics and functions. Studies aimed to identify ASC subpopulations either in targeted approaches via FACS by analysing the expression of specific cell surface markers [17,36,47] or in a broader approach by single-cell RNA-sequencing (scRNA-seq) [12,31,45,48]. This led to the identification of functionally distinct ASC populations and contributed to the ongoing elucidation of an ASC hierarchy.

In the present study, we investigate DPP4 as a functional marker of human ASC subpopulations. DPP4 was previously described as a surface marker for a proliferation competent ASC population with multipotency and relatively low adipogenic differentiation capacity in sWAT of mice [45]. While DPP4^+^ ASCs were detected in WAT depots of young mice [45], this could not be confirmed in adipose tissue depots of middle-aged and old mice by scRNA-seq [12]. In the present study, we identified DPP4^+^ ASC as a highly abundant subpopulation of the proliferation and differentiation competent DLK1^−^/CD34^+^ ASC population [14] by FACS analysis of freshly isolated SVFs from human sWAT. Human DPP4^−^ ASCs have considerably reduced proliferative and self-renewal capacity relative to DPP4^+^ ASCs, while adipogenic differentiation capacity is higher in DPP4^−^ ASCs. These results suggest that DPP4^+^ cells are higher in the stem/progenitor cell hierarchy, while DPP4^−^ cells are progenitors more committed to adipogenic differentiation. Our data further suggest that upon *in vitro* cultivation of ASC routinely isolated by plastic-adherence, the proliferation competent DPP4^+^ ASCs most likely overgrow DPP4^−^ progenitors.

Both DPP4^+^ and DPP4^−^ ASCs display typical ASC gene expression profiles [17], lacking expression of the multipotency markers NANOG, SOX2 and OCT4 [49] but highly expressing markers for somatic stemness, KLF4 and c-MYC [20,50–52], and quiescence, p21^Cip1^, p27^Kip1^ and p57^Kip2^ [53,54]. Moreover, the early adipogenic markers C/EBPβ and C/EBPδ are moderately expressed, whereas the adipogenic key regulator PPARγ2 is only low expressed.

The knock-down of DPP4 decreases the proliferative and self-renewal capacity of ASCs, while adipogenic differentiation is enhanced. Moreover, upon OE of DPP4 in ASC, the adipogenic differentiation capacity is dramatically abrogated. These loss-of-function and gain-of-function experiments support the model that DPP4 facilitates proliferation and stemness and attenuates adipogenesis capacity in ASC. Stemness of ASC is typically characterized by high proliferation, self-renewal and multilineage capacity [24,25,55], and ASC higher in the stem cell hierarchy can be distinguished by lower adipogenic differentiation capacity and higher proliferation and self-renewal capacity from progenitors that are already prone to adipogenesis [4,45]. According to this concept, our results suggest that DPP4 is a marker of ASCs with high stemness playing a crucial role in maintaining the stem cell status of human ASCs.

In the present study, we detect in whole mount staining experiments DPP4 protein at the surface and in many but not all CD34^+^ cells in sWAT localized in the vascular interface surrounding small blood vessels, the typical ASC niche in human sWAT [13–16]. These data underscore that CD34^+^/DPP4^+^ cells comprise a major fraction of human ASCs. Furthermore, we detect DPP4 in mature adipocytes in human sWAT. These data resemble previous studies with human ASC, showing that DPP4 is upregulated in the course of adipogenesis resulting in terminally differentiated adipocytes with high level of DPP4 protein where it is involved in the regulation of insulin signalling [41,43]. It was also shown that the expression of DPP4 in adipocytes of different WAT depots positively correlates with obesity potentially linked to paracrine functions of DPP4 in its soluble form as adipokine [42]. Our finding that DPP4^+^ ASCs display little adipogenic capacity whereas DPP4 protein is present in mature adipocytes suggests that DPP4 might display different functional roles likely depending on cell type and differentiation stage. This is in line with the known role of DPP4 in metabolic diseases as major incretin hormones responsible for postprandial insulin secretion are among the substrates of its peptidase activity [56]. Adipocytes were identified as major sources of circulating soluble DPP4 [42,57] going along with our observation that mature adipocytes in sWAT express DPP4. However, the function of DPP4 in maintaining stemness in ASC seems to be independent from its role as adipokine. DPP4^+^ progenitors with high stemness were identified in WAT of mice [45], and their contribution to *de novo* formation of mature adipocytes has been proven recently [58]. Taken together with our results, these findings suggest differential roles of DPP4 in ASC and adipocytes.

In conclusion, we demonstrate that DPP4 is localized in a population of CD34^+^ ASCs residing in the stromal layer surrounding small vessels and in mature adipocytes of human sWAT. These findings are in accordance with earlier studies, showing that DPP4 is an ASC protein that is induced in the course of adipogenic differentiation and has functions in adipocytes [41]. Additionally, we show that DLK1^−^/CD34^+^/DPP4^+^ ASCs isolated from human sWAT, *ex vivo*, comprise an ASC subpopulation with high proliferative and self-renewal capacity and low adipogenic differentiation potential relative to DLK1^−^/CD34^+^/DPP4^−^ ASC. Moreover, experiments inducing loss-of-function and gain-of-function of DPP4 in ASC suggest that DPP4 is required for the maintenance of proliferation and self-renewal capacity and attenuation of adipogenic differentiation. Thus, our study suggests that DPP4 is a functional marker of an ASC subpopulation with relative high stemness.

## Material and Methods

### Adipose tissue donors

Human sWAT samples were obtained from patients undergoing routine abdominoplastic surgery at the Department for Plastic and Reconstructive Surgery at the Medical University of Innsbruck, Austria. The study has been approved by the ethical committee of the Medical University of Innsbruck according to the Declaration of Helsinki. All donors gave informed written consent. No malignant or severe metabolic disorders were reported for any of the donors. Clinical anthropometric parameters of all donors are listed in Supplementary Table S1.

### Cell culture

Upon cultivation, cells were always seeded in DMEM/F-12 medium with HEPES (4-(2-hydroxyethyl)-1-piperazineethanesulfonic acid) and L-glutamine containing 33 µM biotin, 17 µM pantothenate and 20 µg/ml ciprofloxacin supplemented with 10% FBS, referred to as ASC2 medium. After 16 h, medium was changed to proliferation medium PM4 (DMEM/F-12 medium with HEPES and L-glutamine containing 33 µM Biotin, 17 µM pantothenate, 10 ng/ml EGF, 1 ng/ml FGF, 500 ng/ml insulin and 20 µg/ml ciprofloxacin supplemented with 2.5% FBS). To starve/synchronize ASC before induction of adipogenesis, medium was changed to ASC1 (DMEM/F-12 medium with HEPES and L-glutamine containing 33 µM biotin, 17 µM pantothenate and 20 µg/ml ciprofloxacin without any FBS).

### Isolation of the SVF and ASC from human sWAT

Isolation of the SVF and subsequently of ASC was performed as previously described [25]. Briefly, tissue samples were washed in PBS and cut into small pieces after removing blood vessels and connective tissue. Dissected tissues were incubated for 90 min at 37°C in a digestion buffer consisting of 200 U/ml collagenases (CLS Type I, Worthington Biochemical Corp., Lakewood, NJ) and 2% w/v BSA in PBS. After several purification steps, including filtration, centrifugation and incubation in erythrocyte lysis buffer (0.155 M Na_4_Cl, 5,7 mM K_2_HPO_4_, 0.1 mM EDTA, pH 7.3), SVF was suspended in ASC2 medium. SVF cells were directly subjected to flow cytometric analysis or sorting, or for standard ASC isolation SVF cells were seeded in high density; after 16 h medium was changed to ASC1 medium, and cells were cultured in serum-free medium for 6 d. Then, ASCs were harvested, reseeded and cultured in PM4 medium (DMEM/F-12 medium with HEPES and L-glutamine containing 33 µM biotin, 17 µM pantothenate, 10 ng/mL EGF, 1 ng/mL FGF, 500 ng/mL insulin and 20 µg/mL ciprofloxacin, supplemented with 2.5% FBS) for amplification and used for experiments or frozen and stored in liquid nitrogen.

### Flow cytometry

For flow cytometry analysis, cells were washed in PBS and subjected to immunofluorescence (IF) staining with anti-CD34-PE-Cy7 (BD Pharmingen, #556626), a rat monoclonal anti-human DLK1/Pref1 (AdipoGen, #AG-25A-0091) along with anti-rat-APC (BD Pharmingen, #5510109), anti-CD31-FITC (BD Pharmingen, #555445), anti-CD45-APC-Cy7 (BD Pharmingen, #561863) and anti-DPP4/CD26 (Biolegend, #302715). Cells were measured using a FACS Canto II (BD Bioscience), and data were analysed using FlowJo 10.5.2 software. For *ex vivo* analysis of ASC, flow cytometric sorting of freshly isolated SVFs was performed. Therefore, cells were subjected to IF staining with following antibodies: anti-CD34-PE-Cy7, a rat monoclonal anti-human DLK1/Pref1 along with anti-rat-APC, and anti-DPP4/CD26, and sorted using a FACS Aria (BD Bioscience). For cell count experiments, events recorded during 90 s were assessed, and separate biological triplicates were measured.

### shRNA-mediated DPP4 knock-down and DPP4 overexpression

To achieve DPP4 loss-of-function in human ASC, pLKO.1 vectors containing shRNAs targeting the human DPP4 gene were purchased from a commercial supplier (Dharmacon, Lafayette, LA, USA). Besides a non-targeting control (scr), two shRNAs were used, sh1 (clone Id: TRCN0000050773) and sh2 (clone Id: TRCN0000050774). For DPP4 gain-of-function, a DPP4 overexpression vector was obtained from the DNASU plasmid repository (clone Id: HsCD00829736) and cloned into a pLenti 6.2 V5-DEST vector by Gateway LR recombination. To prepare endotoxin-free plasmids, all plasmids were amplified in Stbl3 *E. coli*, and the Endo Free Plasmid Kit (#12362, Qiagen, Hilden, Germany) was used for plasmid isolation. Lentiviral particles for ASC infection were produced in HEK293 FT cells by preparing DNA-Lipofectamine 3000 (Life Technologies, #L3000015) complexes containing psPAX packaging plasmid, pMD2.G envelope plasmid and the respective p.LENTI expression plasmid. Virus titre was determined in U2OS cells, and particles were stored at −80°C. For infection, 800,000 ASCs were seeded in 175 cm^2^ cell culture flasks in ASC2 medium. On the next morning, medium was changed to PM4, and after another 6 h, ASCs were infected with a multiplicity of infection of 4 by diluting the respective amount of virus in PM4 containing 6 µg/ml polybrene. The virus was applied overnight, and then medium was changed to fresh PM4 for 1 d followed by selection medium for 3 d (2 µg/ml puromycin and 10 µg/ml blasticidin in PM4 for shRNAs and overexpression, respectively).

### Transient transfection of U2OS cells

Transient transfection of U2OS cells with a DPP4 expression vector was done by incubation with DNA-Lipofectamine 3000 complexes for 6 h followed by a medium change. Cells were harvested for further use the following afternoon.

### IF staining

For whole-mount IF staining, sWAT specimens (~0.5 cm^3^) were fixed in Lillie’s neutral 4% formaldehyde for 1 h at RT followed by 4× washing in PBS. After blocking the samples in B-Buffer (PBS, 1% Triton X-100, 0.2% sodium azide, 5% FCS) for 2–3 d at 4°C, tissue probes were incubated with first antibodies in B-buffer for 72 h at 4°C followed by alternating washing in W-buffer (PBS, 1% Triton X-100, 0.2% sodium azide) and PBS 4× each. Next, tissue samples were incubated with conjugated secondary antibodies diluted in B-buffer together with DAPI for 24 h at 4°C. After alternating washing in W-buffer and PBS, and a final washing in W-buffer overnight at 4°C, tissue samples were equilibrated in glycerol for 1 h, placed in a Petri dish with a glass bottom (CELLview Dish, Greiner Bio-One, #627861, Vienna, Austria) and analysed using a confocal laser scanning microscope (Cell Voyager CV1000, Yokogawa, spinning-disc/50 μm pinholes). Images were collected in *z*-stacks with a maximal step-height of 0.6 μm. The primary antibodies used for IF staining were a PerCp-Cy5.5-conjugated mouse anti-human DPP4/CD26 monoclonal antibody (Biolegend, #302715), rabbit anti-human CD34 antibody (Abcam, #ab81289), rabbit anti-human Perilipin (Cell Signaling, #9349). Secondary antibody used for IF staining was goat anti-rabbit Alexa Fluor 488 (Abcam, #150077). Nuclei were stained blue with DAPI (4',6-diamidino-2-phenylindole). Images were acquired in 40-fold magnification using oil immersion. The PerCp-Cy5.5-conjugated mouse anti-human DPP4/CD26 monoclonal antibody was tested by IF stainings of U2OS cells transiently transfected either with a DPP4 overexpression vector or a respective control mock vector (Supplementary Fig. S2A, 2B). Therefore, U2OS cells were seeded on 15 mm cover slides placed in six-well plates, transiently transfected and, upon 70% confluency fixed in 4% PFA, permeabilized in Triton-NaCitrat (0.5% Triton and 0.1% NaCitrat in PBS) with PBS washing steps in between. Subsequently, cells were blocked for 10 min in PBS + 1% BSA and incubated with antibodies for 1 h at RT. After final washing steps in PBS + 1% BSA, cover slides were mounted on slides and analysed using a confocal laser scanning microscope (Cell Voyager CV1000, Yokogawa, spinning-disc/50 μm pinholes). As additional negative control U2OS cells transiently transfected with a DPP4 overexpression vector were stained with an isotypic PerCp-Cy5.5-conjugated monoclonal mouse antibody (anit-CD20, BD, #558021) (Supplementary Fig. S2C).

### Adipogenic differentiation

For adipogenic differentiation, ASCs were grown in PM4 medium to confluence in 6- or 12-well plates. Then, medium was changed to serum-free ASC1 for 48 h for synchronization. Adipogenesis was induced by changing to a differentiation medium (ASC1 supplemented with 0.2 µM insulin, 0.25 µM dexamethasone, 10 µg/ml transferrin, 0.5 mM IBMX (3-isobutyl-1-methylxanthine) and 2.5% FBS (fetal bovine serum). On day 3 post-induction of adipogenesis and subsequently every 2–3 d, medium was changed to differentiation medium without IBMX. For all samples, cells were cultivated in three wells to obtain biological triplicates per donor.

### Triglyceride staining

Triglyceride formation in differentiating ASC was assessed by Oil Red O staining on day 14 post-induction of adipogenesis. Therefore, cells were fixed in 4% paraformaldehyde in PBS for 1 h and stained with 0.5% Oil Red O in isopropanol/water (60:40) for 15 min. For quantification, isopropanol extraction of Oil Red O was performed, and OD (optical density) was measured at 570 nm. Three technical triplicates per donor were obtained.

### BrdU Assay

BrdU incorporation was measured using the 5-bromo-2´-doxy-uridine Labeling and Detection Kit III (Roche, #11444611001, Vienna, Austria) according to the manufacturer´s manual. Therefore, 1600 cells/well were seeded in 96-well plates in ASC2 medium. After 16 h, medium was changed to PM4 ± BrdU (final concentration 10 µM). After 4 and 6 d, cells were fixed, incubated with nuclease working solution, stained with anti-BrdU-POD Fab fragments (final concentration 200 mU/ml) and incubated with POD substrate before absorption at 490 nm was measured. Three technical triplicates per donor were obtained.

### Colony formation assay

As a measure of self-renewal capacity, colony formation assays were performed. Therefore, 1000 cells/well were seeded in six-well plates in ASC2 medium. After 16 h, medium was changed to PM4 and subsequently changed every 2–3 d. Between day 14 and day 21, cells were fixed with freshly prepared methanol/acetone (1:1) for 10 min and stained with crystal violet for 15 min to count colonies. Six technical triplicates per donor were obtained.

### Real-time quantitative PCR

For gene expression analysis, RNA was isolated using the RNeasy Plus Micro Kit (Qiagen, #74034, Hilden, Germany) according to the manufacturer´s instructions, RNA concentration was measured with NanoDrop (Thermo Scientific, Vienna, Austria), and reverse cDNA synthesis was performed using the First Strand cDNA Synthesis Kit (Thermo, #1622, Vienna, Austria). For cDNA synthesis, 200 ng RNA was used, and cDNA was diluted 1:5 for real-time quantitative PCR (RT-qPCR). RT-qPCR was conducted using a QuantStudio^TM^ Flex System (Thermo Fisher Scientific, Vienna, Austria) with AceQ SYBR Green Master Mix (Vazyme, Q111-02, Nanjing, China). All samples were measured in technical triplicates. Primer sequences are given in Supplementary Table S2.

### Protein-level analysis

To analyse protein levels, cells were harvested in SDS (sodium dodecyl sulfate) lysis buffer and sonicated. Protein concentrations were determined using the Compat-Able Protein Assay Preparation Reagent Set (Thermo Fisher Scientific, #23215, Vienna, Austria) and the Pierce BCA Protein Assay Kit (Thermo Fisher Scientific, #23227, Vienna, Austria) according to the manufacturer´s instructions. 20 µg of total protein per sample was separated by SDS polyacrylamide gel electrophoresis and blotted onto PVDF membranes, which were probed with anti-PPARγ (#23215, Cell Signaling), anti-FABP4 (#10004944, Cayman) or anti-DPP4 antibodies (#ab28340, Abcam). Goat anti-rabbit IgG-HRP (#W4011, Promega) and goat anti-mouse IgG-HRP (#W4021, Promega) served as secondary antibodies. Signals were detected using a chemoluminescence detection system, and membranes were stained with Ponceau S for normalization to total protein. Densitometric analysis was done using ImageJ 1.51n (NIH, USA) and Image Lab version 6.0.1 (Bio-Rad, Feldkirchen, Germany).

### Statistics

All results were confirmed in at least three independent experiments. For cell count experiments two-way ANOVA and elsewise unpaired Student’s *t*-test were performed for statistical analysis. In all diagrams, mean ± SEM is shown, and significances are indicated in the following way: *p < 0.05, **p < 0.01 and ***p < 0.001.

## Supplementary Material

Supplemental Material
