# Supplementary material for: Dipeptidyl peptidase-4 cell surface expression marks an abundant adipose stem/progenitor cell population with high stemness in human white adipose tissue
Source: Adipocyte. 2022 Oct 3;11(1):601–15. doi: 10.1080/21623945.2022.2129060 (PMC9542856; doi:10.1080/21623945.2022.2129060)
Supplement: Supplemental Material [file KADI_A_2129060_SM9320.zip › supplement/Supplementary content.docx]

**Supplementary Figure S1. Cell surface expression of ASC marker in the DPP4^+^ and DPP4^-^ ASC subpopulations**

Freshly isolated SVF subjected to cell surface staining. A) Frequencies of DLK1^-^/CD34^+^/DPP4^+^/CD24^-^, DLK1^-^/CD34^+^/DPP4^+^/CD24^+^, DLK1^-^/CD34^+^/DPP4^-^/CD24^+^, and DPP4^-^/CD24^+^ populations in the SVF, and B) representative Dot Blot. n=6 donors, mean ± SEM is shown.

**Supplementary Figure S2. Test detection of DPP4 protein using anti-DPP4/CD26 antibodies by immunofluorescence staining in U2OS cells overexpressing DPP4.**

U2OS cells were transiently transfected with A) a DPP4 overexpression vector or B) a respective control mock vector, fixed and permebealized, stained with DAPI and a PerCp-conjugated mouse anti-human DPP4/CD26 monoclonal antibody and viewed in immunofluorescence experiments by confocal laser scanning microscopy. C) U2OS cells were transiently transfected with a DPP4 overexpression vector, fixed and permebealized, and stained with DAPI and an isotypic PerCp-Cy5.5-conjugated antibody.

**Supplementary table 1. Clinical anthropometric parameters of sWAT donors**

| **donor** | **sex** | **age** | **BMI** | **height (cm)** | **weight (kg)** |
| --- | --- | --- | --- | --- | --- |
| 1 | f | 38 | 24.45 | 168 | 69 |
| 2 | f | 35 | 22.23 | 167 | 62 |
| 3 | f | 29 | 22.99 | 172 | 68 |
| 4 | m | 18 | 24.49 | 183 | 82 |
| 5 | f | 34 | 25.95 | 170 | 75 |
| 6 | f | 21 | 25.04 | 166 | 69 |
| 7 | f | 28 | 22.86 | 162 | 60 |
| 8 | f | 61 | 26.11 | 159 | 66 |
| 9 | f | 50 | 27.99 | 168 | 79 |
| 10 | f | 40 | 24.68 | 172 | 73 |
| 11 | f | 35 | 27.43 | 162 | 72 |
| 12 | m | 27 | 29.24 | 184 | 99 |
| 13 | m | 37 | 25.25 | 178 | 80 |
| 14 | f | 26 | 24.97 | 155 | 60 |
| 15 | m | 62 | 25.64 | 181 | 84 |
| 16 | m | 21 | 25.06 | 173 | 75 |
| 17 | f | 38 | 26.56 | 160 | 68 |
| 18 | f | 41 | 23.14 | 165 | 63 |
