# Supplementary material for: Dipeptidyl peptidase-4 cell surface expression marks an abundant adipose stem/progenitor cell population with high stemness in human white adipose tissue
Source: Adipocyte. 2022 Oct 3;11(1):601–15. doi: 10.1080/21623945.2022.2129060 (PMC9542856; doi:10.1080/21623945.2022.2129060)
Supplement: Supplemental Material [file KADI_A_2129060_SM9320.zip › supplement/supplementary Figures.pptx]

## Slide 1
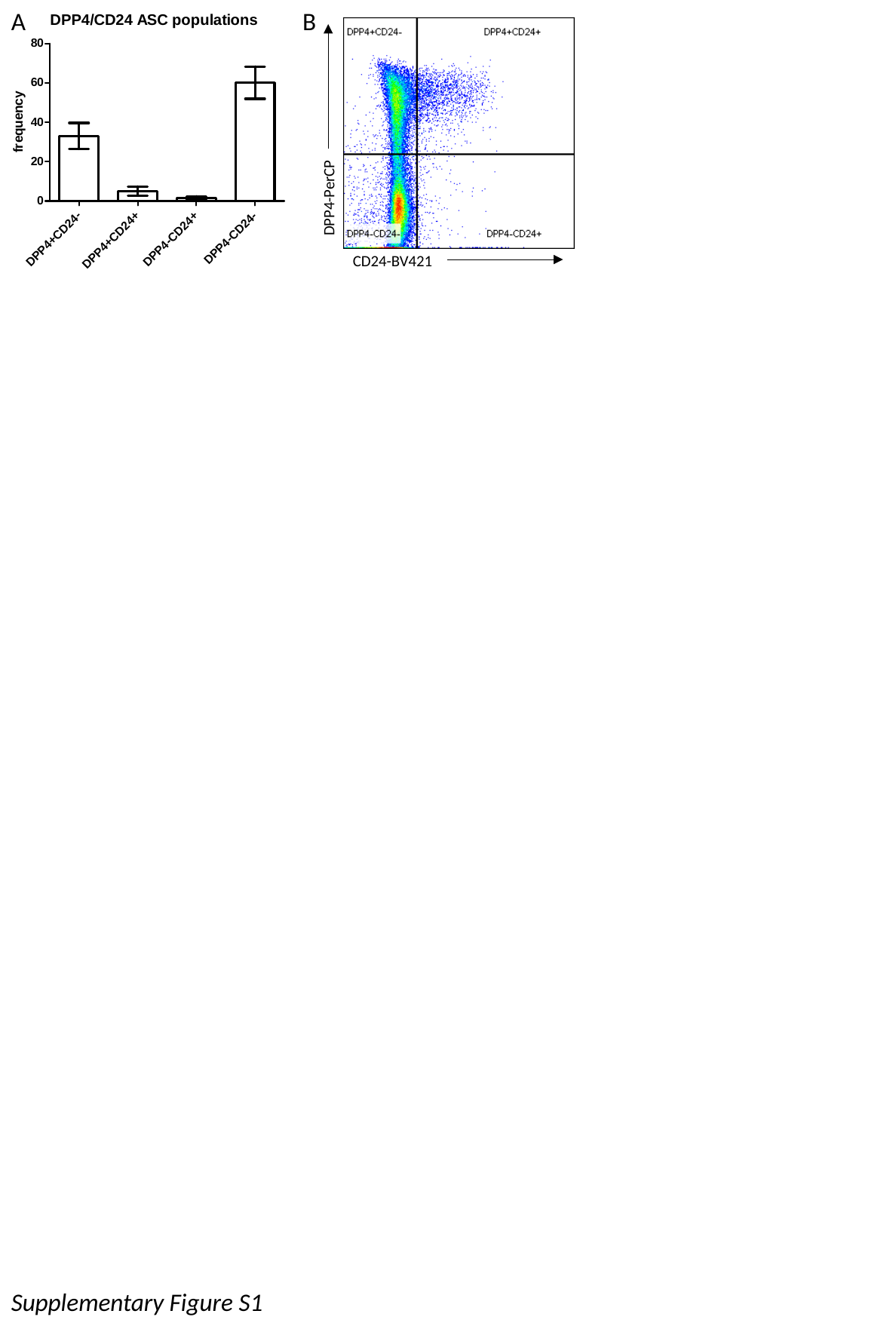

A
B
DPP4-PerCP
CD24-BV421
Supplementary Figure S1

## Slide 2
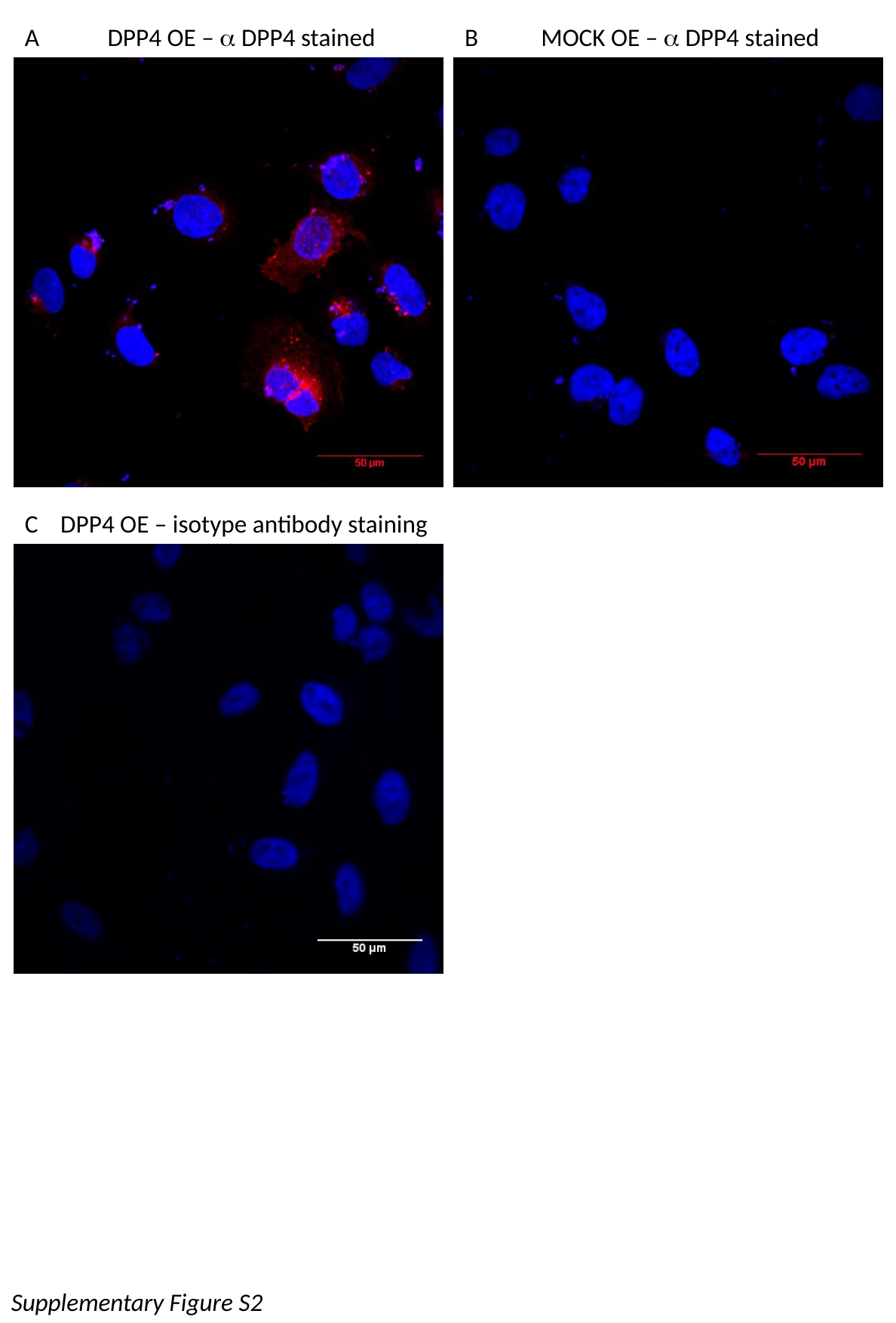

A
DPP4 OE – a DPP4 stained
B
MOCK OE – a DPP4 stained
C
DPP4 OE – isotype antibody staining
Supplementary Figure S2
